# Inbreeding avoidance, competition and natal dispersal in a pair-living, genetically monogamous mammal, Azara’s owl monkey (*Aotus azarae*)

**DOI:** 10.1098/rsos.240379

**Published:** 2024-08-07

**Authors:** Margaret Corley, Alba Garcia de la Chica, Griëtte van der Heide, Marcelo Rotundo, Adalgisa Caccone, Eduardo Fernandez-Duque

**Affiliations:** ^1^ Department of Ecology and Evolutionary Biology, Yale University, New Haven, CT, USA; ^2^ Owl Monkey Project, Fundación ECO, Formosa, Argentina; ^3^ Departamento de Ecología, Genética y Evolución-Facultad de Ciencias Exactas y Naturales, Laboratorio de Ecología y Comportamiento Animal, Universidad de Buenos Aires, Buenos Aires, Argentina; ^4^ Department of Anthropology, University of Colorado Boulder, Boulder, CO, USA; ^5^ Department of Anthropology and School of the Environment, Yale University, New Haven, CT, USA; ^6^ Facultad de Recursos Naturales, Universidad Nacional de Formosa, Formosa, Argentina

**Keywords:** dispersal, inbreeding avoidance, competition, owl monkey

## Abstract

Natal dispersal is an important life-history stage influencing individual fitness, social dynamics of groups and population structure. Understanding factors influencing dispersal is essential for evaluating explanations for the evolution and maintenance of social organization, including parental care and mating systems. The social and mating systems of Azara’s owl monkeys (*Aotus azarae*) are infrequent among mammals; these primates are pair-living, serially and genetically monogamous and both sexes directly care for offspring. To evaluate the role that competition and inbreeding avoidance play in shaping dispersal patterns, we used 25 years of demographic and genetic data to examine how variation in timing of natal dispersal is related to social (adult replacements, step-parents, births and group size) and ecological factors (seasonal abundance of resources) in a wild population of *A. azarae* in Formosa, Argentina. We found that all males and females dispersed from their natal groups, but subadults delayed dispersal when a step-parent of the opposite sex joined the group, indicating that they may perceive these step-parents as potential mates. Dispersal was more probable when resource conditions were better, regardless of age. Overall, agonistic conflict over food and potential mates with adults in the natal group, as well as inbreeding avoidance, contribute to regulating dispersal.

## 1. Introduction

Natal dispersal (hereafter dispersal), the movement of individuals from their birthplace to areas in which they may breed, is a critical process that influences the fitness of individuals, group demography, social dynamics and the genetic structure of groups and populations [1,2]. From the perspective of an individual, dispersal is a behavioural strategy that has major consequences for the individual’s health and survival [3]. Dispersal has profound impacts on social organisms, in particular, as it affects the availability of potential social and mating partners. It thus influences access to reproductive opportunities, and ultimately helps to determine an individual’s fitness [4–6]. At the level of the group, whether individuals disperse or remain in the natal group to breed impacts social dynamics within and between groups, patterns of relatedness and the potential for indirect fitness [7,8]. At the level of the population, patterns of dispersal impact gene flow and community dynamics [1,9,10], ultimately shaping patterns of biodiversity [2]. Documenting the individual, group and population implications of dispersal is essential for explaining the evolution of social and mating systems [11–14], and for effectively managing populations and predicting the consequences that environmental changes may have on species’ distributions [15–17].

The study of dispersal had an initial historical focus on examining the relationships between mating systems and life-history traits to explain variation in patterns of dispersal [11,13,18–20], later enriched with attention to the evolutionary history of the taxa [21–23]. In the last several decades, the ‘genetics’ revolution generated a new set of theoretical and empirical evidence showing that the relationship between dispersal patterns and mating systems is more complex than had traditionally been assumed [2,14,24–26]. These advances led to a variety of new theoretical explanatory models, that look beyond phylogeny or mating systems, to provide adaptive explanations for why, when and how individuals leave their natal groups [27–29].

Over all these years, two main hypotheses have guided the research on the evolution of dispersal. The *inbreeding avoidance hypothesis* states that the evolution of dispersal is driven by the risks of mating with close kin if staying in the natal group [11,13,20,23,30–32]. Inbreeding avoidance is the hypothesis most often invoked for explaining dispersal in pair-living taxa [13,23,32]. In these species, offspring of both sexes lack access to unrelated potential mates and must therefore disperse in order to avoid breeding with close kin. However, animals may not always avoid inbreeding [33,34] and serial monogamy and extra-pair mating can lead to situations in which individuals who have yet to disperse can gain access to unrelated opposite-sex adults [35,36]. The *competition avoidance hypothesis* states that the evolution of dispersal is primarily driven by a need to minimize the fitness detriments of competing with kin for access to mates or resources [11,13,29]. Based on this hypothesis, factors such as group size, the introduction of new (unrelated) individuals to the group, local population density and food availability in the natal territory may influence the relative amount of competition for resources with kin and thus the likelihood of dispersal [37–42].

Evaluating explanations for complex behaviours, such as dispersal, is challenging, especially in wild populations of relatively long-lived taxa. Diverse influences, including individual (e.g. sex, size and body condition), social (e.g. natal group composition, density of mates and/or competitors) and ecological (availability of food, territories, etc.) factors, may all contribute to dispersal decisions [3,29,39]. It is therefore necessary to understand the behavioural ecology, social and mating system of a species, including information on tenure length, patterns of parental care and relatedness within groups [30,43]. Knowing the genetic structure of groups is essential for explaining why individuals of one sex, or both sexes, leave their natal groups; thus, genetic data can provide important insights into general patterns of dispersal and gene flow. However, to determine how social and ecological factors influence dispersal requires multi-year demographic monitoring of identified groups of individuals. Combining demographic information with genetic data on identity and parentage is a powerful means of assessing how relatedness and familiarity influence dispersal decisions, and the effects that dispersal has on individuals, groups and populations.

Here, we leverage demographic, life-history and genetic data to evaluate how well the resource competition and inbreeding avoidance hypotheses explain dispersal patterns in owl monkeys. Dispersal is a multi-phase life-history process that can include emigration (leaving from the natal area), a transient phase (i.e. floating [44]) and immigration (settling in a new area) [9,45], and each of these phases may be under specific selective forces to reduce the overall cost of dispersal [3,25]. In this study, we focus on the first of these phases, the timing of emigration, and examine social and ecological factors that may influence when individuals leave their natal group.

The Azara’s owl monkey (*Aotus azarae azarae*) is a pair-living, monogamous, arboreal primate that exhibits biparental care. The population of owl monkeys in Formosa, Argentina, is composed of social groups consisting of two reproducing adults, which are genetically monogamous [46], and 1–4 young [47]. The population also includes 4–9 solitary floater individuals per square kilometre [44]. Both adult males and adult females are replaced regularly by these floaters; the resulting serial monogamy means that young are frequently living in natal groups with unrelated adults who represent potential mates or mate competitors [48,49]. Analyses of demographic data offered some preliminary findings on dispersal patterns [50,51].

We present here analyses spanning 25 years, during which we monitored the composition and demographic changes of groups, ecological variables and collected biological samples, from which genetic marker data were obtained. We use these data to describe dispersal patterns and evaluate proximate factors that may be influencing variation in the age and timing of natal dispersal in this pair-living primate. We examine how social and ecological variables (i.e. group size, infant births, adult replacements and food availability) contribute to variation in the age and timing of natal dispersal. Specifically, we predict that if inbreeding avoidance is the primary cause of dispersal, then dispersals will consistently occur before, or around, the time individuals reach sexual maturity. Even when serial monogamy sometimes introduces unrelated step-parents to the natal group, these potential mates are not always available to the young; some pairs of reproducing adults maintain their relationship over a decade, indicating that inbreeding avoidance may still be important in this population. If resource competition is an important factor, dispersals will occur more frequently in particular seasons associated with different valuable resources (e.g. mating and food). Since dispersal is costly, owl monkeys will generally disperse during seasons (e.g. spring) when food resources are most abundant. However, offspring may be forced to disperse at less than ideal times of the year when conditions are particularly harsh, if food competition with natal group members becomes more intense. In this case, dispersals will occur during the harsh, winter months, at times when fruit abundance is lower. If mating competition is an important driver, then the timing of dispersals will be influenced by adult replacements that introduce potential mates/mate competitors to the natal group. We predict that the influence of an adult replacement will depend on the age and sex of the offspring, relative to the new adult. If the replacement occurs prior to the sexual maturity of the offspring, we do not predict it to consistently influence the mean age, or timing, of dispersal. However, if a replacement occurs after an offspring begins to undergo sexual maturity (e.g. a subadult), we predict two alternative outcomes: offspring in the presence of a potential mate remain in their natal group longer, in order to retain access to the new potential mate, or they leave the natal group sooner because they are expelled by their same-sexed genetic parent with whom they are now experiencing mate competition.

## 2. Methods

### 2.1. Study site

We studied the natal dispersal of Azara’s owl monkeys (*A. azarae azarae*) at the Reserva Privada Mono Mirikiná, a 1500 ha reserve on a private cattle ranch in the Humid Chaco of Formosa, Argentina. The Owl Monkey Project (OMP) of Argentina has studied this owl monkey population at Estancia Guaycolec (58°13′ W, 25°54′ S) since 1996 [52,53]. Specifically, a 300 ha area of gallery forest along the banks of the Riacho Pilagá has been mapped and groups within this area have been habituated. Owl monkeys (*A. azarae*) at this site live in both the gallery forest and forest patches; the data reported here come from groups residing in the gallery forest, with the exception of one group, Colman, which resides in a xerophytic forest patch next to the core study area. An additional description of the site and methods are provided in the electronic supplementary material.

### 2.2. Data collection

*Demographic data*: To identify changes in group composition (i.e. births, replacements, dispersals and deaths) we used a combination of regular monitoring of identified, habituated groups and information on genetic identity and parentage. Each time we contacted a group, the observer recorded the structure of the group (size and age of each group member) and the identity of each previously identified individual to document changes in group composition since the previous sighting. The unequivocal identification of individuals is facilitated by the presence of bead and radio collars [8,54]. We excluded individuals for which the dates of birth or dispersal could not be estimated with an accuracy of at least six months (i.e. the animal is first noticed as missing more than a year after it was last seen in the group).

Our dataset included 329 births in 29 groups recorded between September 1996 and December 2021 (table 1). Adult replacements were determined from changes in group composition as described above. Regarding ‘dispersals’, we classified the absence of an individual from its natal group between 1997 and 2020 in one of the three categories: presumed dispersal, confirmed dispersal or dead (table 1). A ‘presumed dispersal’ refers to an individual, 18 months or older, missing from its natal group that was not seen again outside of the natal group. We chose 18 months as the minimum age for a potential dispersal because this is the youngest age when an offspring in the study population has been confirmed to leave the natal group and survive independently. We use ‘confirmed dispersal’ for an individual that, after leaving its natal group, we observed ranging solitarily, or living as an adult in a new group. We distinguish between presumed and confirmed dispersal because, while it is reasonable to presume that a missing offspring >18 months has dispersed, it is still possible that some of them may be missing because they died while still in their natal groups. We classified an individual less than 18 months old as ‘dead’ when missing from its natal group; those 18 months or older were considered ‘dead’ when observers could identify the remains of a particular individual within the home range of the natal group.

**Table 1.** Births and fates (presumed dispersal, confirmed dispersal, death, still in natal group or unknown) of offspring born in each of 30 social groups of Azara’s owl monkeys (*A. azarae*) at the OMP study site in Formosa, Argentina between 1996 and 2021.

group	years with birth records	births				offspring fates				
		total births	U	M	F	presumed dispersals	confirmed dispersals	deaths	still in group	unknown
A500	1998–2021	13	13	0	0	10	1	0	2	0
A900	1997–2021	13	11	2	0	5	1	2	3	2
B68	1997–2021	16	11	4	1	8	2	5	1	0
C0	1997–2021	15	8	3	4	10	3	1	1	0
camp	1997–2005	6	3	3	0	2	1	3	0	0
CC	1996–2021	17	8	3	6	5	5	5	2	0
Colman	1999–2021	18	11	3	4	7	3	6	2	0
Corredor	1999–2021	14	10	1	3	8	1	5	0	0
D100	1996–2010	5	3	1	1	3	1	1	0	0
D1200	1996–2021	20	14	3	3	11	3	4	2	0
D1400	2003–2011	4	4	0	0	0	0	0	0	4
D500	1996–2021	20	8	4	8	8	5	7	0	0
D800	1996–2021	13	9	4	0	6	2	4	1	0
E350	1996–2021	17	8	4	5	7	2	7	1	0
E500	1997–2021	18	7	5	6	6	5	4	3	0
Aranda	2012–2013	2	2	0	0	0	0	1	0	1
F1200	1997–2021	18	11	2	5	8	4	4	2	0
F700	2000–2021	16	10	3	3	6	5	3	2	0
Fauna	2001–2010	8	6	1	1	4	1	2	0	1
G1300	2001–2021	10	9	0	1	3	1	5	0	1
H900	2005–2021	4	3	1	0	2	0	0	1	1
I200	2003–2004	2	2	0	0	0	0	0	0	2
IJ500	1997–2021	13	10	1	2	6	1	5	1	0
L100	1998–2020	12	11	1	0	11	0	0	1	0
Morito	2002–2004	2	2	0	0	1	0	0	0	1
P300	2001–2021	13	10	1	2	8	1	4	0	0
Parrilla	2001–2007	5	4	1	0	3	0	2	0	0
Picada C.	2000–2020	9	6	1	2	6	0	1	1	1
Soldado	2003–2008	4	4	0	0	0	0	0	0	4
Veronica	2000–2020	10	7	2	1	6	1	1	2	0
total	1995–2021	337	225	54	58	160	50	82	27	18

‘Years with birth records’ is the span of years during which a given group was monitored and all births that occurred during this range were recorded.

‘Presumed’ and ‘confirmed’ dispersals are defined in the text. ‘Deaths’ includes both confirmed and presumed deaths (all disappearances of individuals at <18 months). ‘Unknown’ refers to individuals for whom we were unable to determine the category to which they belonged (owing to gaps of >2 years between sightings of their natal group).

F, female; M, male; U, sex unkown.

*Sampling*: Biological material (i.e. ear punches, blood or hair) was collected from 173 unique individuals during captures; still, most samples used for DNA analyses presented here were obtained non-invasively by recovering faeces from the forest floor immediately after an identified animal defecated (*n* = 602 faecal samples). We used these samples to determine the genetic sex of dispersing individuals [55] and to establish genetic relationships among group members using microsatellite genotypes.

*Microsatellite data collection:* To assess identity and parentage we developed a panel of 22 microsatellite loci by screening the genome for *A. azarae* made available to us in advance by J. Rogers and colleagues [56]. From this screening, we identified 50 potential variable loci, all from different contigs, for which sequences with M-13 tails on forward primers were designed [57]. Primers for these 50 candidate loci were tested with a subset of 20 DNA extracts from owl monkeys sampled in Formosa, Argentina. We selected 22 loci that amplified consistently and showed variation in the study population for subsequent analyses used to identify individuals and parentage (electronic supplementary material, table S1). Microsatellite genotypes were used to establish identities of uncaptured individuals and establish genetic relationships among group members (i.e. to determine if pre-dispersing young were living with genetic parents or with one or more unrelated step-parents).

*Ecological data*: There is a strong seasonal pattern in the forest’s productivity, with the availability of owl monkey food sources reaching a nadir during the winter dry season (June–August). For analyses of the relationship between resource competition and dispersal, we used counts of fruit from the four most important dry season fruit-producing species, to create an index of winter fruit availability for each year in each owl monkey group’s home range. Specifically, we used phenological data, collected monthly from approximately 300 trees in plots located in the core of the OMP’s study area from 2003 to 2021 [58,59] (see electronic supplementary material for details).

### 2.3. Data analysis

**Figure 1.**
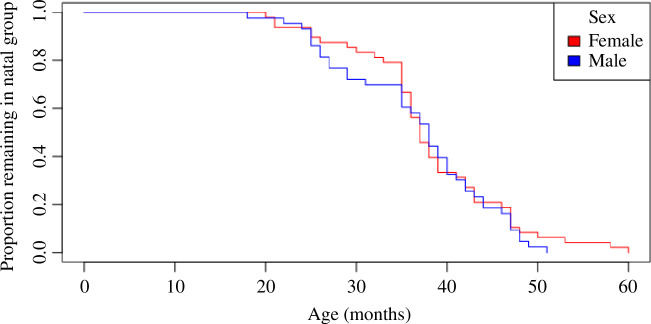
Proportion of individuals with either presumed or confirmed dispersals remaining in the natal group, from birth to 60 months, for owl monkeys of known genetic sex: 48 females (red) and 43 males (blue).

**Figure 2.**
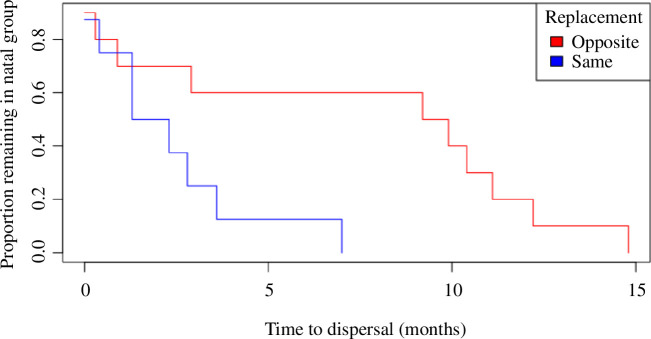
Proportion of subadults with presumed or confirmed dispersals that left the natal group after experiencing a replacement of the same-sex (blue) or opposite-sex (red) parent (*n* = 19).

We calculated summary statistics for the age at dispersal and the time to dispersal after an adult replacement. We also constructed ‘survival curves’ to show the proportion of individuals remaining in the natal group at a given age for confirmed and presumed dispersals (electronic supplementary material, figure S1), for male and female dispersers (figure 1), and for the time to dispersal for subadults that experienced adult replacements (figure 2). We summarized the seasonality of dispersal by calculating the proportion of dispersals that occurred in each month and in each season (electronic supplementary material, figure S4).

When modelling our data, we conducted three sets of analyses. Set 1 examines the relationship between social composition of the natal group and age at dispersal. Set 2 investigates the relationship between adult replacements and time to dispersal. Set 3 explores the relationships between social and ecological factors and the seasonality of dispersal. For each of the three, we took an information theoretic approach [60,61] and defined an *a priori* set of models (Cox regression models for Sets 1 and 2; generalized linear regression models for Set 3). We chose explanatory variables (table 2) for inclusion in the models considering their biological relevance, based on previous knowledge of owl monkey dispersal, and social dynamics in other pair-living and socially monogamous taxa [12,49,62,63]. Tables with additional details and summaries of our data and model are presented in the electronic supplementary material. Datasets and code used in our analyses are reposited on FigShare [64]. Requests to use raw data for research purposes can be made to E.F.-D.

**Table 2.** Name, type and description of the variables used in each part of the owl monkey dispersal analyses using statistical models.

variable name	type of variable	analyses set	description
explanatory variables			
group size	count	1, 3b	number of individuals in the group (range 2–6) at the time of the dispersal, NOT including the individual dispersing
time since last birth	continuous numerical	1	number of months the dispersing individual was in the natal group since the last birth in the group (equal to the age of the dispersing individual if there were no younger offspring born prior to dispersal)
adult replacement	categorical	1, 3	whether a dispersing individual had experienced the replacement of an adult prior to the dispersal (yes or no)
sex	categorical	2	sex of the dispersing individual (male, female or unknown)
age at replacement	continuous numerical	2	age (months) of the dispersing individual when the replacement occurred
same-sex replacement	categorical	2	whether the adult replaced was the same sex as the dispersing individual (yes or no)
dispersal age	continuous numerical	3	age (months) when the dispersal occurred
recent birth	categorical	3a	whether there was a birth in the natal group within the past 12 months (yes or no)
winter fruit abundance	continuous numerical	3	fruit availability in the winter dry season (May– August) in the year that an offspring dispersed
outcome (dependent) variables			
age at dispersal	continuous numerical	1	age (months) when an individual permanently dispersed from the natal group
time to dispersal	continuous numerical	2	time (months) after an adult replacement that an individual remained in the natal group before dispersing
birth season	categorical	3a	whether an individual dispersed during the birth season (mid-September–early January) or outside this season
winter season	categorical	3b	whether an individual dispersed during May–August (the period of low resource abundance)

The first set of analyses examined general patterns of dispersal and how the ‘age of dispersal’ is related to competition over resources, evaluated through the social composition of the natal group. For this analysis, we chose ‘group size’, ‘time since last birth’, and ‘adult replacement’ as potential explanatory variables (table 2). We included group size and time since last birth because they may influence the amount of resource competition within a disperser’s natal group. Since owl monkeys are pair-living, variation in group size is largely determined by the presence of infants or younger offspring. Adult replacements also are likely to influence social dynamics, particularly between adults and older individuals (juveniles and subadults), since non-related individuals can become competitors for food resources and present mating opportunities [12]. We did not include sex because age at dispersal is fairly similar for males and females [50,65] and sex was known for only 34% of offspring in the dataset (*n* = 112/325); thus, not including sex helps avoid overfitting. We used these variables to construct seven candidate Cox-proportional hazard models that considered censored data to explain the variation in age at dispersal (electronic supplementary material, table 2*a*). We removed individuals who died prior to reaching the minimum dispersal age (*n* = 75) and ran the seven candidate models on the remaining dataset (*n* = 173 individuals), censoring individuals still in their natal groups (*n* = 22). We also wanted to examine the effect that our method of categorizing dispersals, specifically including offspring that disappeared (i.e. presumed dispersal), may have on estimating age at dispersal. To do this, we ran the seven candidate models again using only confirmed dispersals (*n* = 46) (electronic supplementary material, table 2*a*).

The second set of analyses explored the potential influence of mate competition and inbreeding avoidance on dispersal by examining how adult replacements, which introduce a potential mate or mate competitor to the group, may influence the timing of dispersal. When a solitary floater displaces an adult in a group and becomes the new resident adult it probably changes social interactions between adults and offspring. Whether this new adult represents a potential mate, or a mate competitor, will depend on the young’s age and sex [12,66]. Thus, we included ‘age at replacement’ and ‘same-sex replacement’ as potential explanatory variables. Females begin to develop mature hormone profiles prior to dispersing [67], but it is unknown if males become sexually mature while still in the natal group. We therefore also included the disperser’s sex in interaction terms in this set of models. We constructed nine Cox-proportional hazard candidate models (electronic supplementary material, table 2*b*) to examine how explanatory variables related to how long an offspring remains in the natal group after experiencing a replacement (‘time to dispersal’). We used data from the subset of individuals who experienced a replacement and had dates of birth and dispersal that could be estimated with an accuracy of at least six months (58 individuals: 32 presumed dispersals and 26 confirmed dispersals). Some individuals experienced more than one adult replacement. Since an individual can only disperse once, we only considered the one replacement that occurred most immediately before dispersal.

Since an adult replacement exposes offspring to an unrelated adult, older offspring who are undergoing sexual maturity (i.e. subadults) may perceive, or be perceived by, this new adult as a potential mating partner or mating competitor. We thus predicted that adult replacements would influence subadults differently from younger offspring. To test this prediction, we performed a separate analysis that included only the subset of individuals who were subadults (>24 months old) at the time of the replacement and whose genetic sex was known. Twenty-two individuals experienced, as subadults, the replacement of their same-sex parent (*n* = 4), opposite-sex parent (*n* = 8) or both parents (*n* = 7); the sex of three subadults whose sex was unknown were excluded from analyses. To avoid overfitting the data, we did not create candidate models for this comparatively smaller subset of data. Instead, we calculated the mean number of months that individuals who experienced the replacement of a same, or opposite, sex parent remained in the natal group after the replacement.

The third set of analyses investigated the influence of resource competition on the seasonality of dispersal. To attempt disentangling the potential influences of group composition changes associated with the birth season [68] and resource abundance we defined seasonality in two ways and performed separate analyses on each. First, we constructed 15 candidate generalized linear models for which the outcome variable was whether a dispersal occurred during the ‘birth season’, with a dataset of 210 individuals. ‘Age at dispersal’, ‘group size’, ‘adult replacement’ and ‘recent birth’ were included as potential explanatory variables (table 2; electronic supplementary material, table S2*c*). We did not use time since last birth, as in the other two sets of analyses, because births, being highly seasonal, would have been correlated with the outcome variable. Second, we constructed 15 candidate models for which the outcome variable was whether dispersal occurred during the dry ‘winter season’, when resource abundance and temperatures are lowest [69]. The ‘dry season’ of May–August is a period of limited food availability [59]; groups occupy territories that provide reliable dry season foods within the core areas [58]. Given that when offspring disperse they lose reliable access to resources in their natal group’s core home range, these months of low food availability and low temperatures probably represent the time when dispersing and subsequently ranging solitarily is most difficult. We therefore designed our candidate models to examine which variables might explain why individuals sometimes disperse during the harshest time of the year. As potential explanatory variables, we included ‘dispersal age’, ‘adult replacement’, ‘group size’ and an estimate of food availability during the dry season, ‘winter fruit abundance’ (table 2; electronic supplementary material, table S2*c*). Our dataset for these models included 109 individuals from groups for which we were able to estimate winter fruit availability in the natal group’s home range.

We performed statistical analyses in R v. 3.3.2 [70], using the package AICcmodavg (v. 2.1-0) to calculate model characteristics and model-averaged parameter estimates, their standard errors and 95% unconditional confidence intervals (table 3) [71]. We report the mean ± 1 s.d. unless otherwise specified.

**Table 3.** Model-averaged estimates for the three sets of analyses examining age at dispersal (Set 1), time to dispersal after an adult replacement (Set 2) and the seasonal timing of dispersal (Set 3) in owl monkeys. See table 2 for units and additional information about each variable.

variable	coefficient estimate [95% CI]^a^	standard error	HR estimate [95% CI]^b^	analyses set
group size	−0.30 [−0.54 to 0.06]	0.12	0.74 [0.58 to 0.94]	1
time since last birth	−0.02 [−0.04 to 0]	0.01	0.98 [0.96 to 1]	1
adult replacement	−0.35 [−0.69 to −0.01]	0.17	0.70 [0.50 to 0.99]	1
age at replacement	0.09 [0.05 to 0.12]	0.02	1.1 [1.05 to 1.12]	2
same-sex replacement	0.75 [0 to 1.49]	0.38	2.12 [1 to 4.42]	2
adult replacement	0.45 [−0.16 to 1.08]	0.32	1.57 [0.85 to 2.92]	3a
dispersal age	0 [−0.03 to 0.03]	0.02	1 [0.97 to 1.03]	3a
recent birth	−0.54 [−1.19 to 0.11]	0.33	0.58 [0.30 to 1.12]	3a
group size	0 [−0.4 to 0.4]	0.02	1 [0.67 to 1.49]	3a
adult replacement	−0.57 [−1.61 to 0.47]	0.53	0.57 [0.20 to 1.60]	3b
winter fruit abundance	0 [0 to 0]	0	1 [1 to 1]	3b
group size	−0.49 [−1.15 to 0.17]	0.34	0.61 [0.32 to 1.19]	3b
dispersal age	0 [−0.05 to 0.05]	0.03	1 [0.95 to 1.05]	3b

^a^ 95% unconditional confidence interval for the model-averaged coefficient for each explanatory variable in each set of models.

^b^ Hazard ratio with 95% confidence interval.

## 3. Results

### 3.1. Float on: both males and females always disperse and avoid reproducing in their natal groups

Dispersal was an obligatory life-history stage. No individuals reproduced in the group where they were born (*n* = 337, 30 groups, 1997–2020, table 1). They all dispersed from their natal groups or died before having reproduced there. Of 319 offspring whose fates could be determined, 66% dispersed (*n* = 50 confirmed and *n* = 160 presumed), 26% died (*n* = 82) and 8% (*n* = 27) were still in their natal groups at the end of the study.

The age at dispersal was highly variable. Offspring whose age at dispersal was estimated to ±6 months dispersed when they were between 1.5 and 5 years old (18–60 months; median = 35 months; mean ± s.d. = 34.5 ± 9.1 months). Hence, some offspring dispersed before the onset of sexual maturity, while others stayed well after it [67].

Males and females did not differ much in their ages of dispersal (median (mean ± s.d.): males = 38 (36.6 ± 8.5) months, females = 37 (38.0 ± 8.7) months). Even though both sexes had similar probabilities of dispersing at most ages, the probabilities of dispersal were somewhat male-biased at earlier (28–35 months) and latest (50–60 months) ages (figure 1).

### 3.2. Size and composition of the natal group are not strongly associated with earlier dispersals

Dispersing individuals (*n* = 210) experienced variation in the size of their natal group, whether step-parents were present (i.e. a replacement had occurred), and whether there was a recently born sibling at the time they dispersed. The mean size of the natal groups was 4.2 ± 0.8 s.d. (range 3–7) including the dispersing individual, 31% (64/210) of them had lived with at least one step-parent, and a mean of 11.1 ± 9.3 s.d. months (range 0–46) had passed since the last infant was born in the natal group.

The time since an infant was born (‘last birth’) was included in all four models that met the selection criteria (electronic supplementary material, table S2*a*). Specifically, an additional month since the last birth decreased the monthly chance of dispersal by 2% as indicated by the −0.02 model-averaged estimated effect of this variable (table 3). Having a step-parent in the group before dispersal (‘adult replacement’) and ‘group size’ were each included in two of the four models. Young in groups with step-parents had 35% less chance of dispersing at a given age than those who lived with their biological parents; and having a larger group size at the time of dispersal reduced the chance of dispersal by 30% (table 3). Confidence intervals (95% CI) for the hazard ratio of all three variables approach or include 1, indicating that evidence for the influence of these variables was not very robust. Sensitivity analyses conducted with a more conservative subset of the data (i.e. including only confirmed dispersals) also produced hazard ratios with 95% CI that included 1 (electronic supplementary material, table S3).

### 3.3. Mating competition: subadults are less likely to disperse if a potential mate joins the group

Losing a biological parent did not increase the probability of dispersal. About a third (31%, *n* = 64) of the 210 dispersing individuals were, at the time of dispersal, living with at least one step-parent. This situation occurred because one, or both, of their biological parents had been evicted from the group by a same-sex solitary floater [48]. Age at dispersal was similar for individuals living with at least one step-parent and those who had both biological parents present at the time of dispersal (mean ± s.d. for step-parent(s) versus biological parents: 36.1 ± 9.5 months versus 34.7 ± 8.7 months; *n* = 111). A more conservative analysis limited to confirmed dispersals produced similar results (39.8 ± 9.2 versus 39.7 ± 6.3 months; *n* = 45) (electronic supplementary material, figure S2). There was much variation in the temporal relationship between losing a biological parent and dispersing. Individuals left the natal group 0–39.7 months after their parent had been evicted (mean ± s.d.: 16.0 ± 10.5 months; 13.6 ± 9.5 months if only the most recent replacement is considered for individuals who experienced multiple replacements). There was little difference in the mean age at dispersal for those who had lost their biological mother (36.9 ± 9.2 s.d.) or father (36.4 ± 8.5 s.d.); and in the time to dispersal since the replacement (mothers versus fathers: 14.5 ± 10.7 months versus 17.5 ± 10.1 months; electronic supplementary material, figure S3*a*). This was also true when only subadults (age >24 months) were considered (subadults remained 6.3 ± 4.3 and 5.9 ± 6.0 months, after male and female replacements, respectively; electronic supplementary material, figure S3*b*).

**Figure 3.**
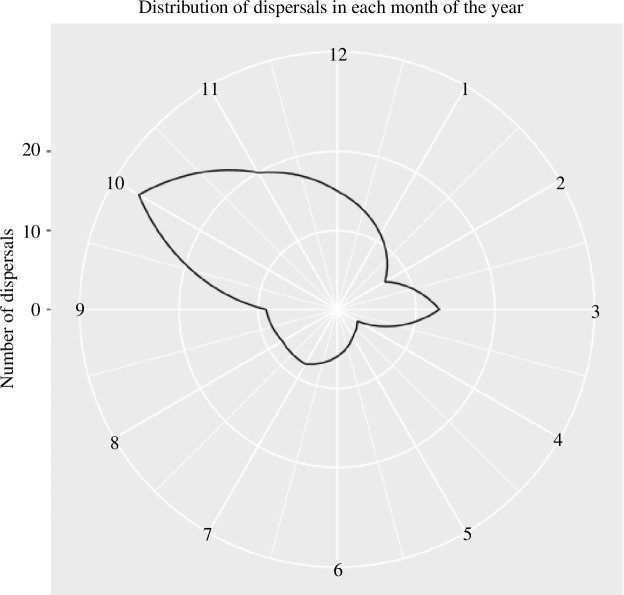
Seasonal distribution of dispersals from 1997 to 2021 for 133 owl monkeys (*A. azarae*) with confirmed or presumed dispersals. The white circles indicate the number of dispersing individuals, with the centre indicating 0 and each concentric circle indicating an increase by 10 (scale is shown on the left side). The numbers around the outer circle indicate the month of the year (January–December), and the solid black line indicates the number of individuals dispersing in each month.

The models indicate that the age when an individual experienced a replacement and the sex of the adult being replaced, relative to the individual, were important factors for explaining the time that offspring remained in the natal group. ‘Age at replacement’ was the only variable included in all five models that met the criteria to explain the probability of dispersal if a potential mate joins the group, and it was the only variable included in the best model (electronic supplementary material, table S2*b*). Specifically, the hazard of dispersing increased 1.1 times for each additional month of age at the time a new adult moved into the group. Whether the new adult was of the ‘same sex’ as the dispersing individual, or not, was in three of the five best models. Offspring who experienced the replacement of a parent by a potential mate remained in the natal group longer, as indicated by the averaged model estimates (HR = 2.12; table 3). The sex of the dispersing individual was present only as an interaction term, but not on its own, in any of the five models and did not explain much variance in time to dispersal (electronic supplementary material, table S2*b*).

While the sex of a subadult, on its own, did not explain much of the variance in time to dispersal, the sex of the dispersing individual, relative to the sex of the parent that was replaced, was strongly associated. Specifically, the timing of a subadult’s dispersal varied in relation to whether the new adult in the group was a potential mate (i.e. opposite sex as the subadult) or a potential mate competitor (i.e. same sex as the subadult). Individuals who lived with a step-parent as subadults (i.e. >24 months; *n* = 19) dispersed later when their opposite sex-parent had been replaced by a potential mate than when the same-sex parent had been replaced (mean months to dispersal ± s.d.: 7.2 ± 5.5 versus 2.3 ± 2.2; figure 2). Furthermore, 7 of the 8 subadults that had their same-sex parent replaced dispersed within four months, while more than half (6/10) of those that experienced the replacement of an opposite-sexed parent with a potential mate remained in the natal group for more than nine months (figure 2).

### 3.4. Resource competition: dispersal is more likely when food conditions are better

Dispersals occurred in all months. Still, they were concentrated during the austral spring season (93/210; figure 3; electronic supplementary material, figure S4). They occurred more frequently in spring than expected if they had randomly occurred throughout the year (44% versus 32%, proportion test = *χ* ^2^ = 6.99, d.f. = 1, *p*‐value = 0.008; 95% CI: 0.03–0.22). They peaked markedly in October (*n* = 33), happening nearly twice as frequently than expected by chance (16% versus 8%). Only 29 individuals dispersed in the harsh late-autumn/winter period (May–August), accounting for 20% of dispersals, compared with the 34% expected by chance (*χ* ^2^ = 7.52, d.f. = 1, *p*‐value = 0.006; 95% CI: −0.24 to −0.041; prop 1: 0.20; prop 2: 0.34). They were the least frequent in April and May (figure 3). These seasonal patterns of dispersal, which were similar for males and females (electronic supplementary material, figure S4*a*), also held when the analyses were done on only confirmed dispersal (electronic supplementary material, figure S4*b*).

Age at dispersal was very similar for those who dispersed in the spring season or some other time (mean ± s.d.: 34.7 ± 8.8 months versus 35.9 ± 12.9 months, *n* = 210), and in the harsh winter or in some other time (34.6 ± 12.0 months versus 34.8 ± 8.9 months, *n* = 148; electronic supplementary material, figure S5).

Models examining which variables may be related to dispersing during the spring season, when group composition usually changes and environmental conditions are more favourable, also indicate that age at dispersal is not strongly associated with the seasonality of dispersal. None of the four explanatory variables were strongly associated with spring season dispersals (table 3). ‘Adult replacement’ increased the chance of dispersing during the birth season 1.6 times, but the 95% confidence intervals for this coefficient estimate included 1. If a birth had occurred in the natal group within the past year, the chance of dispersing during the birth season decreased by 60%, though the 95% confidence intervals for this hazard ratio estimate also included 1. While age at dispersal and group size were each included in at least 2 of the 5 models selected for averaging (electronic supplementary material, table S2*c*), both variables had averaged coefficients near 0, and corresponding hazard ratios of 1, indicating that neither was important in explaining whether dispersals occurred in the spring season.

The prediction that resource competition influences dispersal is not supported. The mean age at dispersal was very similar for individuals who dispersed in the winter and those who did it at other times of the year (mean ± s.d., age at dispersal: 34.6 ± 8.9 months versus 34.8 ± 12.0 months; electronic supplementary material, figure S5). Likewise, the mean amount of winter fruit available during the year of dispersal was also similar for individuals dispersing during winter and in other seasons (electronic supplementary material, figure S6). In the models, winter fruit abundance and age at dispersal explained almost none of the variability of winter season dispersals (table 3), as both had hazard ratios of 1. Of the 15 candidate models that met the selection criteria, only seven accounted for more than 5% of the cumulative weight (electronic supplementary material, table S2*c*).

In agreement with previous results on the influence of having an unrelated adult in the group on the probability of dispersal during the birth season (mid-September to early January), models indicated that individuals who lived with an unrelated adult were 37% less likely to disperse between May and August. Though the 95% confidence intervals for the hazard ratio for adult replacement included 1, replacements were substantially less frequent for individuals dispersing in winter than for those doing it in other seasons (32% (8/28) versus 42.5% (48/118)). On the other hand, more individuals dispersing in the birth season (35.9%, 33/92) had experienced a replacement than those that dispersed outside the birth season (26.7%, 31/116).

## 4. Discussion

Even when there was much variation in the age and timing of owl monkey dispersal one finding was very consistent across generations and 25 years of observations. Both males and females always dispersed and they never reproduced in their natal groups.

We predicted that if inbreeding avoidance was the primary cause of dispersal, then dispersals would consistently occur before, or around, the time individuals reach sexual maturity (approximately 3 years). The variation in age at dispersal indicates that while some individuals of both sexes dispersed around the time they reached sexual maturity, others delayed dispersal for up to 2 years [67,72]. While 31% of individuals had an unrelated adult (i.e. a potential mate) replace a genetic parent, the remaining 69% resided in natal groups with only close kin until they dispersed. These findings imply that, even when serial monogamy occurs, inbreeding is still a risk for the majority of owl monkeys who remain in the group after having reached sexual maturity. Thus, inbreeding avoidance does not, on its own, explain well the timing or age of dispersal in owl monkeys, but it is important in explaining why all individuals dispersed prior to reproducing.

We predicted that if mating competition was an important driver of dispersal, then the timing of dispersals would be influenced by adult replacements, but that the context of these replacements (i.e. age when they were experienced by the offspring) would be important [12]. As predicted, we found that adult replacements that occurred while offspring were young (<24 months), did not seem to be associated with earlier age at dispersal. Similarly, if the age when an offspring experienced an adult replacement was not taken into consideration, then having an adult of either sex replaced by a step-parent was not associated with leaving the natal group sooner (table 3). In contrast, for older offspring who had already undergone, or were close to the age of sexual maturity (i.e. subadults), the introduction of an unrelated step-parent was associated with the timing of dispersal, though this sample size was relatively small (*n* = 19). Specifically, when the replacement introduced an adult of the opposite sex (a potential mate), then owl monkeys remained in their natal groups longer than when the replacement introduced an adult of the same sex (a potential mate competitor) (figure 2). This result could signify that owl monkeys may be delaying dispersal to retain access to a new potential mate. However, this decision exposes them to mate competition with their same-sex genetic parent and we did not observe any successful cases of reproduction with step-parents. An alternative explanation is that owl monkeys may remain in their natal group longer when an opposite-sex parent is replaced because their remaining same-sex genetic parent may be more tolerant of them, compared with groups in which the same-sex parent is replaced. If mating competition with kin was driving dispersal, we would expect to see the opposite pattern (subadults dispersing sooner when the opposite-sex parent is replaced). Since owl monkeys disperse sooner after a same-sex adult enters their natal group, mating competition with new, unrelated adults that enter the group seems to play a greater role in triggering dispersal than mating competition with kin.

It is also important to note that just because subadults do not disperse (i.e. are not kicked out by their same-sex parent) when a potential unrelated mate is introduced, this does not mean that they are not being perceived as a potential mate competitor by their same-sex parent. Adults may tolerate subadults and allow them to remain in the group, but use behavioural mechanisms to prevent them from actually engaging in sexual behaviours (i.e. behavioural reproductive suppression). Specifically, adults may mate guard their partner, or act aggressively towards subadults, to prevent them from mating with the new unrelated individual [73,74]. It is also possible that a new adult entering the group may not perceive the subadult as a high-quality mating partner and prefer to mate with the resident adult over the subadult. In this case, the subadult may not actually be perceived by the same-sex parent as a viable mate competitor.

Owl monkey dispersals are highly seasonal, mostly occurring during the spring months when food abundance is high and temperatures are relatively warm (figure 3). This hints at individuals timing their dispersals to begin ranging solitarily, and looking for a mating position, when conditions are mild. October, in particular, may be an ideal time for individuals to disperse, because it is at the beginning of the spring/rainy season, giving individuals the maximum amount of time to float and attempt to acquire membership in a new group while the thermoregulatory challenges of being a solitary are minimal [69]. On the other hand, the variables associated with resource competition that we predicted to influence whether owl monkeys dispersed in winter, outside of this ‘ideal’, mild season, did not explain much of the variation. Resource competition with natal group members should be most severe during the winter, the harshest time of the year. Thus, if resource competition influenced dispersal, we would expect individuals to disperse more frequently during months when less food is available (i.e. when perceived resource competition is most intense). The prediction is not supported by our analyses: winter fruit abundance explained almost none of the variability of winter season dispersals (table 3). Owl monkeys’ dietary flexibility may allow them to shift to consuming more leaves, or other food items, when fruit abundance is lower, which might explain why we did not find evidence of increased food competition or more dispersals during harsher winters. This is consistent with previous work documenting that owl monkey groups’ home ranges produce consistent food resources, even during harsh winters [58].

Age of dispersal, group size or recent births did not explain much of the variability in the seasonality of dispersals (table 3). Both the intensity of food competition and the costs of dispersing and ranging solitarily vary throughout the year, as food availability and climatic conditions change. If, as we predicted, age, through its relationship to a larger body size [75], influenced the level of food competition with natal group members and/or how costly it would be for dispersers to leave the natal group/territory, then we would expect the season in which an individual dispersed to be strongly associated to the disperser’s age. However, age at dispersal was very similar for those who dispersed in the spring season or some other time and in the harsh winter or in some other time (table 3; electronic supplementary material, figure S5).

Thus, overall, the third set of models did not provide much insight into why certain individuals may disperse outside of the expected dispersal season (spring/birth period), or why some even disperse during the harshest months (May–August). The results of these models are supported by plots (electronic supplementary material, figures S5 and S6) showing that dispersers had similar means and levels of variation for age and winter fruit abundance, whether or not they dispersed within the birth season or winter season. Adult replacements may play some role in explaining seasonality of dispersal: our third set of models indicated that adult replacement was associated with a greater likelihood of dispersing during the birth season and a reduced likelihood of dispersing during the winter, but confidence intervals for this effect were large (table 3). It remains unclear why experiencing an adult replacement may make an individual more likely to disperse during the spring/birth season, when conditions are likely most favourable for newly dispersed individuals to range solitarily. One possibility is that the introduction of a non-related adult reduces the nepotistic benefits of philopatry [76,77]. If so, offspring that reside with genetic parents may continue to delay dispersal, owing to potential philopatric benefits associated with remaining near close kin [78,79], whereas those that experience the replacement of a genetic parent with an unrelated step-parent may choose to disperse when conditions are relatively good.

### 4.1. Limitations and future directions

As is the case with all data analyses, we had to make decisions about which variables to include and which subsets of individuals to use in the models, which may have influenced our results. In all cases, we used prior knowledge of owl monkey biology and social systems to perform quality control on the data and select the most biologically relevant variables for the candidate models. Nonetheless, it is likely that variables we were unable to measure, such as body condition or the small-scale abundance of resources, including foods other than fruit, may be important in explaining at least some of the variation in dispersal that we observed.

As with many studies of dispersal, we were limited by our ability to follow individuals who dispersed outside of our study area, which may have influenced our findings. To evaluate the robustness of our results, we performed several sensitivity analysis exercises (electronic supplementary material). For example, including censored individuals still in their natal group, or individuals who had disappeared (presumed dispersals), rather than limiting analyses to confirmed dispersals, had little influence on the magnitude of the effect or hazard ratio and never changed the sign of the estimated effect. Likewise, estimates of our models examining potential causes of winter dispersals (Set 3b) were very similar when we included all individuals and when only the subset of offspring that remained in their natal group until the onset of winter were included (electronic supplementary material, table S3).

We did not include information on what happened to individuals after they dispersed in the analyses. This was because these data are even more challenging to obtain and therefore were not available for the majority of dispersing individuals. However, knowing the fates of individuals who disappear from their natal groups could be important for evaluating biological and evolutionary interpretations of the variation in age and timing of dispersal that we observed. Including presumed dispersals did result in a somewhat earlier mean age at dispersal, compared with the estimate when only confirmed dispersals were considered (electronic supplementary material, figure S1). This suggests that at least some of the individuals who we presumed to have dispersed may actually have disappeared because they died while still in their natal groups, rather than successfully dispersing. While there was a lot of variation in the age and time of year of dispersal, it may be the case that there is an optimal range of ages, or ecological conditions, for it (i.e. individuals who disperse at ideal times or under optimal conditions have a much greater chance to successfully find a breeding position and reproduce). Exploring how the age and timing of dispersal are associated with the amount of time spent as a floater before finding a mate, and ultimately with life-time reproductive success, are important future avenues of investigation. Finally, all the data in our study came from a single location, where territories appear to be saturated. It is unclear to what extent the patterns we observed may be generalizable to other taxa living in different ecological conditions. Future studies that include additional populations, or other *Aotus* species in different environments, would allow us to better assess associations between dispersal patterns and ecological variation through a comparative approach [73].

In summary, in Azara’s owl monkey dispersal is a definite life event, but its timing is flexible. To some extent, inbreeding avoidance and competition over food and potential mates in the natal group all appear to contribute to regulating dispersal, though the relative importance of each factor is context dependent. Our results emphasize that the avoidance of inbreeding, mate competition and resource competition are non-exclusive: these factors may work together to shape the patterns of dispersal.

## Data Availability

All data files and scripts associated with analyses reported in this paper are available in the FigShare repository, which can be accessed with the following link: https://figshare.com/s/a97eb4b3ef9cb5c66d55. Supplementary material is available online [80].
